# Structure-aware graph learning predicts RNA editability across tissues and species

**DOI:** 10.21203/rs.3.rs-8793265/v1

**Published:** 2026-03-19

**Authors:** Zohar Rosenwasser, Michael Levitt, Erez Levanon, Gal Oren

**Affiliations:** Bar-Ilan University, Israel; Stanford University, United States; Bar-Ilan University, Israel; Stanford University, Technion United States

## Abstract

Programmable A-to-I RNA editing using endogenous ADAR enzymes is emerging as a therapeutic strategy, but editability remains difficult to predict because ADAR recognition depends on double-stranded RNA geometry and stability rather than sequence alone. We present ADAREDIT, a structure-explicit graph-attention framework that represents each dsRNA substrate as a nucleotide graph with backbone and base-pair edges and augments this representation with typed interactions and a motif-sensitive sequence branch. We trained and evaluated the model on high-confidence inverted *Alu* duplexes (*n* = 905) with secondary structures predicted by RNAfold and editing levels measured across 8,603 GTEx RNA-seq samples spanning 47 tissues. Across five tissue contexts and comprehensive cross-tissue transfer experiments, ADAREDIT consistently outperformed sequence-only CNN, transformer, and RNA language model baselines and achieved strong discrimination on combined tissue data (AUROC/AUPRC = 0.96; F1 ≈ 0.90). The same graph representation transferred to evolutionarily distant non-*Alu* species (sea urchin, acorn worm, and octopus), indicating conserved principles of ADAR substrate recognition. Finally, attention profiles and *in silico* mutagenesis recapitulated known biochemical constraints, including suppression by an upstream guanosine, and revealed longer-range asymmetric structural influences on editing. The sources of this work are available at our repository: https://github.com/Scientific-Computing-Lab/AdarEdit

## 1 Introduction

Adenosine-to-inosine (A-to-I) RNA editing catalyzed by ADAR enzymes (Adenosine Deaminase Acting on RNA) represents a critical post-transcriptional mechanism that expands proteomic diversity and regulates immune responses Bass (2002); Nishikura (2010); Eisenberg & Levanon (2018). ADAR enzymes selectively deaminate adenosines within double-stranded RNA (dsRNA) structures, converting them to inosines that are interpreted as guanosines during translation. In mammals, ADAR1 and ADAR2 exhibit different tissue-specific expression patterns and substrate preferences, with ADAR1 predominantly editing repetitive *Alu* elements in untranslated regions Levanon et al. (2004); Bazak et al. (2014), while ADAR2 targets mainly specific coding sequences with functional consequences Tan et al. (2017); Nishikura (2016). Dysregulated editing has been implicated in cancer Han et al. (2015); Silvestris et al. (2019), neurological disorders Kawahara et al. (2004); Srivastava et al. (2017), and autoimmune conditions. Beyond its role in various biological pathways, ADAR presents a promising avenue for therapeutic RNA modification. Programmable ADAR-based editing platforms leverage synthetic guide RNAs to direct endogenous ADAR enzymes to specific target sites, enabling site-directed A-to-I conversion for correcting pathogenic mutations Qu et al. (2019); Merkle et al. (2019); Reautschnig et al. (2025). The success of such strategies, however, critically depends on understanding the sequence and structural features that govern ADAR substrate selection.

Despite extensive characterization of ADAR biochemistry, computational prediction of editing sites remains challenging due to the complex interplay between sequence context and RNA secondary structure (Appendix A). ADAR substrate recognition requires both local sequence motifs – including the well-established 5’ neighbor preference against guanosine Eggington et al. (2011); Lehmann & Bass (2000) – and global structural features such as stem length, loop geometry, and base-pairing stability. Early computational approaches relied on manually engineered features including structural parameters (loop length, stem stability, distance to junction) and sequence motifs (nucleotide composition, k-mer frequencies), achieving moderate accuracy Ouyang et al. (2018); Liu et al. (2021). The emergence of deep learning introduced convolutional neural networks that learned sequence patterns automatically, exemplified by EditPredict Wang et al. (2021), which processes linear sequence windows to classify editing sites. More recently, transformer architectures have been applied to RNA editing prediction: Helix Cao et al. (2025) employs a structure-aware attention mechanism that integrates base-pairing probability matrices directly into transformer layers, achieving Spearman correlations of 0.84 for on-target editing; previous work evaluated RNA-FM (a masked language model trained on 23 million sequences Chen et al. (2022)) fine-tuned for editing prediction, and ADAR-GPT Rosenwasser et al. (2026) (continual fine-tuning of GPT-4o-mini on 201-nucleotide sequence windows), achieving competitive performance (F1=0.763 for ADAR-GPT) on liver tissue data. However, while Helix incorporates secondary structure through attention mechanisms, these approaches – including EditPredict, RNA-FM, Helix, and ADAR-GPT – ultimately operate on linear sequence representations rather than explicit graph-based encodings of dsRNA topology, limiting their ability to fully capture the relational nature of base-pairing that determines ADAR binding and catalytic efficiency.

Graph neural networks (GNNs) offer a principled framework for encoding RNA structure Wu et al. (2020); Zhang et al. (2021); Bongini et al. (2022) by representing molecules as graphs where nucleotides are nodes and relationships – both sequential backbone connectivity and Watson-Crick/wobble base pairing – are edges. This representation naturally captures the dsRNA topology essential for ADAR recognition while enabling models to learn multi-scale patterns spanning local nucleotide motifs to global stem-loop architecture. Here, we introduce ADAREDIT, a biology-aware graph attention network Velickovic et al. (2017) that integrates sequence and structure through explicit graph construction from RNA secondary structure predictions. Unlike sequence-only models, ADAREDIT encodes base-pairing relationships and stem-loop geometry directly into the graph, allowing attention mechanisms to weight the importance of structural neighbors dynamically. We further enhance the model with a parallel sequence branch – a convolutional neural network that captures local motifs known to influence ADAR substrate selection – and fuse graph and sequence representations for final classification. Trained on high-confidence Alu-derived substrates across multiple human tissues and evolutionarily distant species, ADAREDIT achieves superior performance (F1 > 0.85) compared to convolutional, transformer, and LLM-based baselines, generalizes robustly to unseen tissues and non-mammalian organisms, and provides mechanistic interpretability through attention analysis that recapitulates known ADAR determinants while revealing novel structural motifs.

### Research questions.

Motivated by the structure-dependent biochemistry of ADAR editing, this work asks:

**RQ1. Does RNA structure provide decisive predictive signal beyond sequence alone?** In other words, when predicting A-to-I editing, how much is gained by explicitly modeling the double-stranded RNA geometry rather than treating RNA as a linear string?

**RQ2. How context-specific is RNA editing across tissues and ADAR environments?** To what extent do editing determinants learned in one tissue transfer to others, and what does the pattern of successes and failures reveal about tissue/isoform specificity?

**RQ3. Are editing determinants conserved across deep evolutionary distances?** If a model learns from human substrates, does it still recognize editability in organisms that lack primate *Alu* elements, suggesting shared structural principles?

**RQ4. Can a high-performing predictor also explain *why* a site is editable?** Can the model recover known biochemical constraints and, at the same time, surface longer-range structural influences that are hard to see from sequence-centric views?

**RQ5. Does learning from abundant repetitive substrates translate to functional targets?** Can models trained primarily on *Alu*-derived duplexes generalize to well-studied protein-coding editing sites in transcripts, supporting practical prioritization for therapeutic design?

### Contributions.

**Figure 1:**
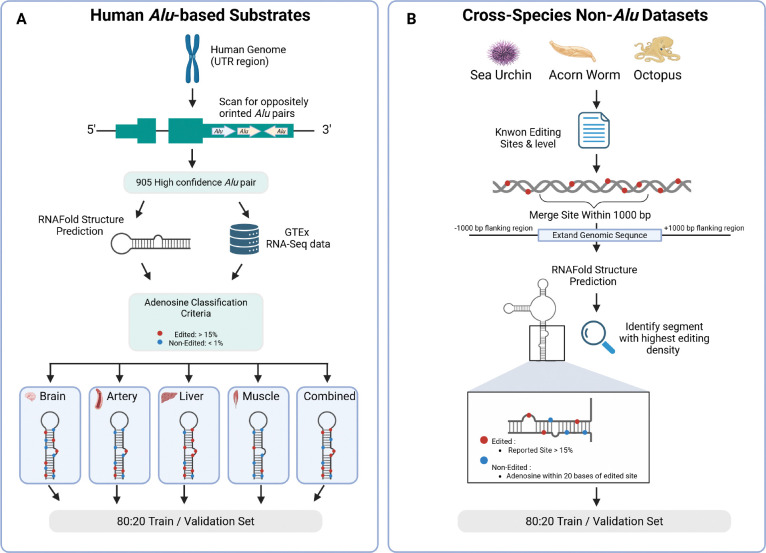
Schematic overview of data construction pipelines. (A) Construction of human *Alu*-derived datasets. Stable dsRNA substrates were identified from inverted *Alu* pairs in UTRs (*n* = 905). For each pair, the secondary structure was predicted using RNAfold. Editing levels were obtained from GTEx RNA-seq data across varying tissues. Adenosines were classified as edited (≥15% level, ≥100 coverage, red) or non-edited (<1% level, ≥100 coverage, blue), creating five tissue-specific datasets where labels vary according to tissue-specific measurements while the sequence and structure remain constant. (B) Construction of cross-species non-*Alu* datasets (sea urchin, acorn worm, octopus). Known editing sites were clustered within 1,000 bp and extended with flanking regions. Following RNAfold structure prediction, the segment with the highest density of sites was selected for each region. Adenosines were labeled as edited based on reported levels (red), whereas unreported adenosines located within 20 bp of an edited site were labeled as non-edited (blue). Both pipelines concluded with an 80:20 train/validation split.

**Figure 2:**
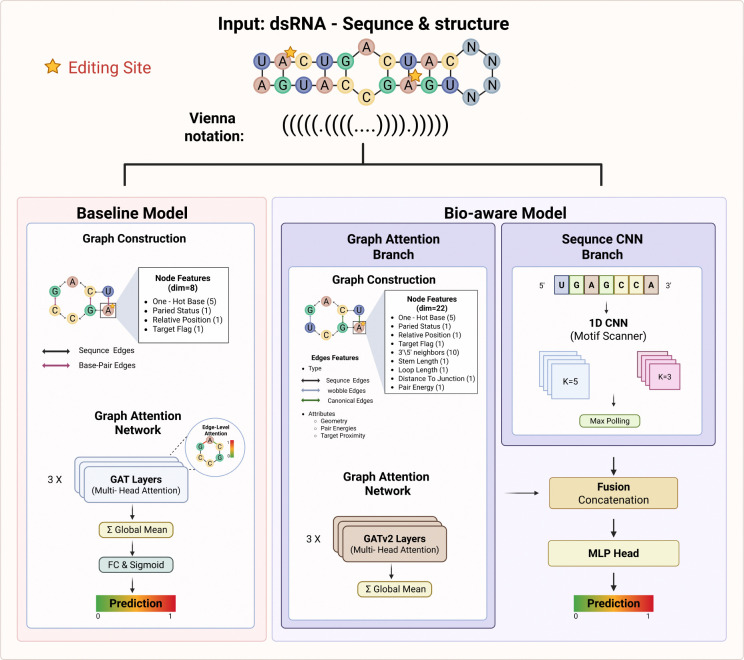
AdarEdit architecture comparing baseline and bio-aware graph neural networks for RNA editing prediction. The figure illustrates the complete workflow for RNA editing site prediction using graph-based representations of double-stranded RNA structures. Top: Input representation showing dsRNA sequence and secondary structure with the candidate editing site (marked with star) and corresponding Vienna dot-bracket notation. Left (Baseline): Graph representation with 8-dimensional node features (base identity, pairing status, relative position, target flag) and edges connecting sequential neighbors (black) and base pairs (purple). Three GAT layers with multi-head attention, followed by global mean pooling and classification. Right (Bio-aware): Two-branch architecture: (1) Graph attention branch with enriched 22-dimensional node features (base, neighbors, geometry, energies) and typed edges (sequence/wobble/canonical); (2) Sequence CNN branch with 1D convolutions (K=3, K=5) capturing local motifs. Graph and sequence embeddings are fused through an MLP head for final prediction.

**Figure 3:**
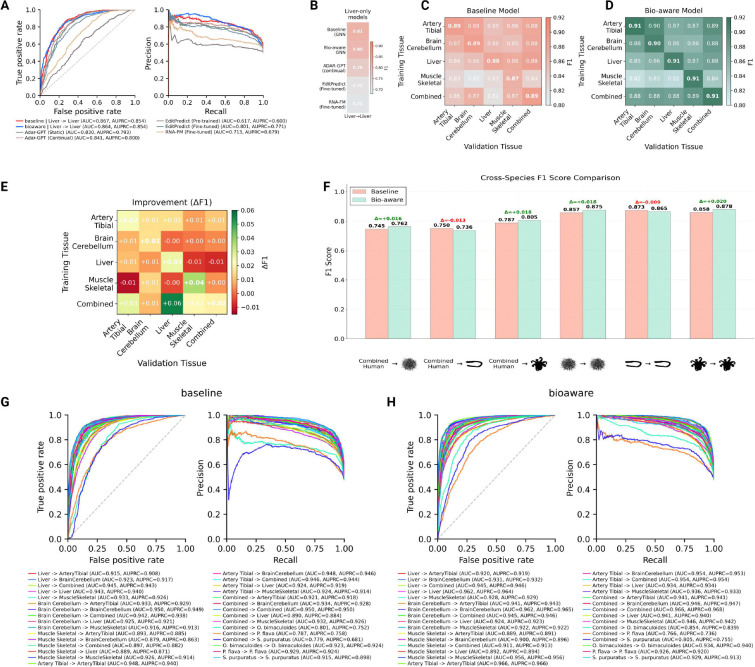
Performance and generalization of graph-based RNA editing prediction models. (A) Receiver operating characteristic (ROC; left) and precision–recall (PR; right) curves comparing ADAREDIT models trained on liver tissue with sequence-only baselines (EditPredict, RNA-FM, ADAR-GPT). Graph-based models show higher AUROC and AUPRC than sequence-only approaches. (B) F1 score comparison for liver-trained models, highlighting performance differences between sequence-only baselines and graph-based architectures. (C,D) Cross-tissue F1 score heatmaps for the baseline (C) and bio-aware (D) ADAREDIT models. Rows indicate training tissue and columns indicate validation tissue. Diagonal entries correspond to within-tissue evaluation; off-diagonal entries quantify cross-tissue generalization across distinct ADAR expression contexts. (E) Heatmap of F1 score differences (ΔF1) between the bio-aware and baseline models for all train–validation tissue combinations. (F) Cross-species F1 score comparison for (i) within-species training and evaluation (species→species) and (ii) transfer from the human Combined model to non-*Alu* species (Combined→species), shown for Strongylocentrotus purpuratus, Ptychodera flava, and Octopus bimaculoides. Bars denote baseline (pink) and bio-aware (green) performance; ΔF1 values are annotated. (G,H) ROC (left) and PR (right) curves for all cross-tissue and cross-species train–validation combinations for the baseline (G) and bio-aware (H) models, illustrating model discrimination across diverse training and evaluation settings.

**Figure 4:**
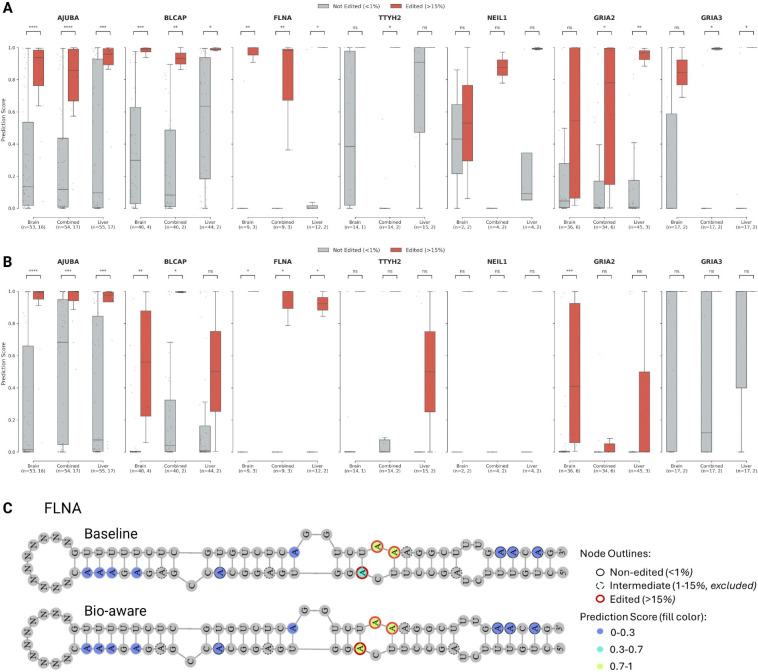
Generalization from *Alu* elements to protein-coding editing targets. (A) Baseline model predictions on seven well-characterized coding region targets across three tissue contexts (Brain Cerebellum, Combined, Liver). Box plots show prediction score distributions for edited sites (>15% editing, red) and non-edited sites (<1% editing, gray) within predicted dsRNA structures. Each target tested with corresponding tissue-trained *Alu* model. Sample sizes (n=edited, non-edited) indicated below each condition. Statistical significance: ns (not significant), *p<0.05, **p<0.01, ***p<0.001, ****p<0.0001. (B) Bio-aware model predictions on the same targets and tissues. (C) Example dsRNA structure from FLNA showing prediction annotations from *Alu* Combined-trained models. Top: baseline model predictions; bottom: bio-aware model predictions. Node outlines indicate editing level status: solid black = non-edited (<1% editing), dashed black = intermediate (1–15% editing, excluded from analysis), red = edited (>15% editing). Node fill colors indicate model prediction score: dark blue (0–0.3), cyan (0.3–0.7), green (0.7–1.0).

**Figure 5:**
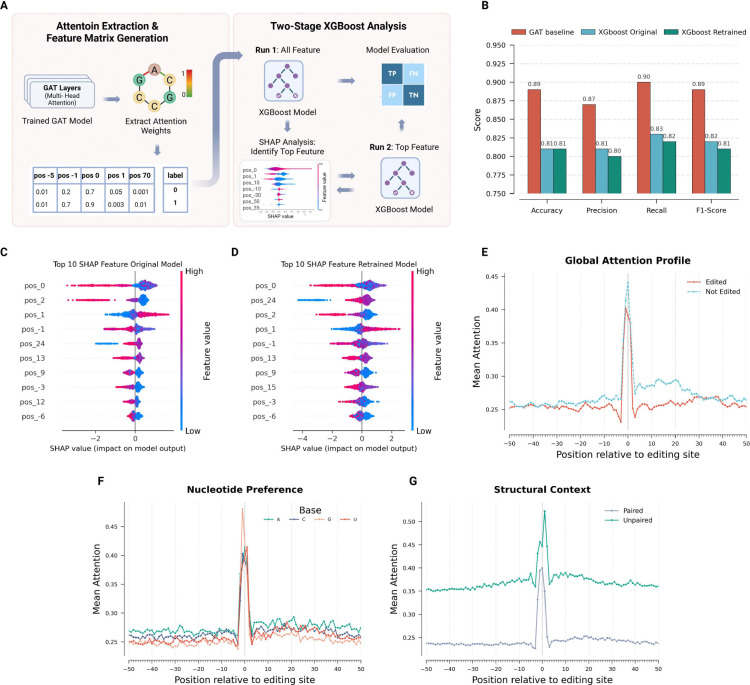
Attention analysis of the baseline model. (A) Interpretability workflow: extraction of edge-level attention weights from the first GAT layer, aggregation into positional features, training of XGBoost classifier on attention features, SHAP analysis to identify top features, retraining with top 20 features, and repeated SHAP analysis. (B) Performance metrics (accuracy, precision, recall, F1-score) for three models: baseline GAT (red), XGBoost trained on all attention features (blue), and XGBoost retrained on top 20 SHAP features (green). (C,D) SHAP summary plots showing top 10 attention features for original XGBoost model (C) and retrained model (D), with feature importance values and direction of influence. (E) Mean attention across ±50 nucleotide windows for edited sites (orange) and non-edited sites (cyan). (F) Mean attention stratified by nucleotide identity (A, U, G, C) at each position relative to the editing site. (G) Mean attention comparing unpaired/loop positions (cyan) versus base-paired positions (green) across the ±50 nucleotide window.

**Figure 6:**
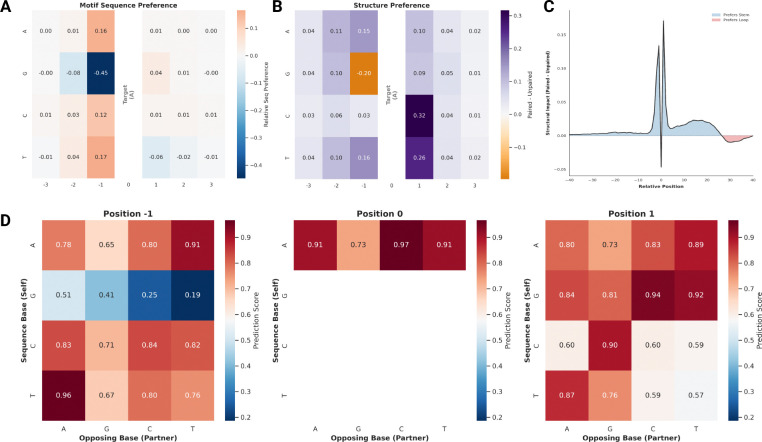
In silico mutagenesis of baseline models reveals sequence and structural preferences. (A) Sequence preference heatmap for positions −3 to +3. Rows show bases A, G, C, U/T; values are normalized prediction scores (position-wise mean subtracted). Red/orange indicates enhanced editing; blue indicates suppression. Position 0 (target A, marked by vertical line) excluded from mutations. (B) Structure-coupled mutagenesis heatmap showing ΔPrediction (Paired - Unpaired) for each base at each position. Purple/dark indicates preference for paired state; orange indicates preference for unpaired state. (C) Positional structural impact across −40 to +40 window. Line shows mean ΔPrediction (Paired - Unpaired); blue shading indicates positions preferring stem structure, red shading indicates positions preferring loop structure. (D) Base-pairing interaction matrices at positions −1, 0, and +1. Each 4 × 4 heatmap shows prediction scores for all combinations of sequence base (rows) and opposing partner base (columns). Higher values (dark red) indicate stronger editing predictions. Position 0 shows only A row as target must be adenosine.

This paper makes the following contributions:

- **Complementary human and cross-species datasets for rigorous evaluation.** Constructs high-confidence evaluation resources spanning five human tissue contexts with distinct ADAR expression profiles and three evolutionarily distant non-*Alu* species, enabling systematic assessment of tissue-specific learning and cross-species transfer (Figure 1).
- **Structure-explicit prediction framework.** Introduces ADAREDIT, a graph-based learning formulation that represents RNA as a structured substrate with both backbone continuity and base-pairing relationships, aligning prediction with ADAR’s structural mode of recognition (Figure 2).
- **Biology-aware modeling, without sacrificing end-to-end learning.** Proposes a biology-aware variant that injects biochemical and geometric cues into the representation while retaining a unified trainable model, and couples this with a complementary sequence branch to capture short-range motif effects (Figure 2, right panel).
- **Robust empirical gains across tissues and baselines.** Demonstrates consistent improvements over strong sequence-only predictors, and systematically characterizes within-tissue performance and cross-tissue transfer to reveal tissue/isoform dependence (Figure 3, Table 1 (Appendix B)).
- **Cross-species transfer as evidence for universal principles.** Shows that structure-aware models retain predictive power when transferred to evolutionarily distant species lacking *Alu* elements, indicating that learned signals are not merely *Alu*-specific (Figure 3F,G,H).
- **Generalization to functional protein-coding targets.** Demonstrates transfer from *Alu*-trained models to well-characterized coding-region editing sites, with differential performance revealing ADAR isoform specificity (Figure 4).
- **Interpretability through attention analysis and structural perturbation.** Uses attention-based analysis and in silico perturbations to identify patterns consistent with established biochemical rules and reveal longer-range structural dependencies, supporting hypothesis generation about editing determinants (Figure 5, Figure 6).

## 2 Data construction

We constructed two complementary datasets to evaluate graph-based RNA editing prediction:

### Human *Alu*-derived substrates (Figure 1A):

Following the protocol established in previous work Rosenwasser et al. (2026), we systematically identified stable dsRNA substrates by scanning all human untranslated region (UTR) for the closest pairs of oppositely oriented *Alu* elements, yielding 905 high-confidence *Alu* pairs. For each pair, we predicted secondary structure using RNAfold Lorenz et al. (2011) and extracted editing levels from GTEx RNA-seq data (8,603 samples across 47 tissues) Lonsdale et al. (2013). Each training/validation example corresponds to a single adenosine position within a predicted dsRNA duplex. Adenosines were classified as edited (≥15% editing level, ≥100 read coverage) or non-edited (<1% editing, ≥100 reads), with intermediate sites (1–15%) excluded to maintain binary classification. We created five tissue-specific datasets – Brain Cerebellum, Artery Tibial, Liver, Muscle Skeletal, and Combined (integrating all tissues) – where the RNA sequence and predicted structure remain identical across tissues, but editing levels and resulting classifications vary according to the measured editing levels from GTEx in each tissue. Each example in the human *Alu* datasets corresponds to one adenosine position within a predicted dsRNA duplex. The data were split 80:20 into training and validation sets, and our split explicitly excludes any adenosine positions used for training from the corresponding validation set, preventing the trivial case where the same site is evaluated twice. Each tissue- specific dataset was class-balanced by downsampling to achieve equal numbers of edited and non-edited examples (50% each class), with the 80:20 split applied after balancing. Dataset sizes and class balance are detailed in Table 1 (Appendix B).

### Cross-species non-*Alu* datasets (Figure 1B):

To test generalization beyond human *Alu* elements, we constructed datasets for three evolutionarily distant species lacking *Alu* repeats: sea urchin (*Strongylocentrotus purpuratus*), acorn worm (*Ptychodera flava*), and octopus (*Octopus bimaculoides*). For each species, we obtained documented editing sites and their measured editing levels from Zhang et al. Zhang et al. (2023). We merged editing sites located within 1,000 bp into discrete clusters, then extracted an extended genomic sequence spanning each cluster plus 1,000 bp flanking regions on both sides. For each extended sequence, we predicted the minimum free-energy secondary structure using RNAfold Lorenz et al. (2011), identified the segment containing the highest density of editing sites, and extracted all adenosines within this segment. Adenosines reported as edited sites (≥15% editing, ≥100 read coverage) were labeled as edited, while all other adenosines within 20 bases in either direction from any edited site were labeled as non-edited. This proximity-based selection ensures that non-edited adenosines have sufficient sequencing coverage – as they are located near confirmed editing sites – yet were not reported as edited, providing high-confidence negative examples. Each species dataset was class-balanced by downsampling to equal numbers of edited and non-edited adenosines, then split 80:20 into training and validation sets.

## 3 AdarEdit Model

### A conceptual gap in modeling RNA editing

Most computational approaches to A-to-I RNA editing have operated on linear representations of RNA, either by modeling sequence windows directly or by augmenting them with engineered descriptors. This is not due to a lack of recognition that structural features matter – the importance of A:C mismatches, dsRNA stability, and stem-loop geometry has long been acknowledged – but rather because structural information is often incorporated as local annotations or summary features rather than as an explicit representation that preserves the relational topology of base pairing. However, ADAR-mediated editing is inherently a *structural* phenomenon: it depends on the formation, stability, and geometry of double-stranded RNA, rather than on sequence motifs alone.

As a result, prior models face an inherent limitation that is not merely architectural, but conceptual: learning from linearized representations can obscure the relational nature of base pairing, where nucleotides distant along the backbone become direct neighbors in the duplex and jointly shape ADAR access and activity. This motivates a structure-native representation that can directly encode both sequential and base-pairing relationships within a unified input.

We developed two graph neural network architectures to investigate the contribution of biology-aware feature engineering to RNA editing prediction. Both models represent RNA duplexes as graphs where nucleotides are nodes and edges encode both sequential connectivity and base-pairing relationships predicted from secondary structure (Figure 2). Each example corresponds to a single candidate adenosine, marked as the target node within the predicted duplex graph.

### Baseline graph architecture

The baseline model employs a streamlined feature set (Figure 2, left panel): each node is encoded with an 8-dimensional vector comprising one-hot base identity (A, G, C, U, N), a binary pairing status flag, relative distance from the candidate editing site (normalized by sequence length), and a target site indicator. Edges are constructed between sequential neighbors (i-to-i+1 backbone connectivity) and between base-paired nucleotides identified by parsing dot-bracket notation from RNAfold structure predictions Lorenz et al. (2011), creating a bidirectional graph that captures both linear sequence context and long-range structural constraints. Three stacked Graph Attention Network (GAT) layers with multi-head attention (4 heads per layer) process this graph, with each layer applying ReLU activation, batch normalization, and dropout (rate=0.2) for regularization. The attention mechanism dynamically learns which neighboring nucleotides – whether sequential or structurally paired – contribute most to editing prediction at each layer. After three rounds of message passing, global mean pooling aggregates node embeddings across the entire graph into a fixed-size representation, which is passed through a fully connected layer followed by sigmoid activation to produce the binary editing probability. Critically, this baseline architecture explicitly encodes RNA secondary structure topology, distinguishing it fundamentally from sequence-only models, but treats all base-pairing interactions uniformly without biochemical differentiation.

### Bio-aware model enhancements

The bio-aware model extends the baseline architecture by incorporating explicit biochemical knowledge at multiple levels (Figure 2, right panel). First, we distinguish edge types according to their biochemical roles: sequential edges connecting backbone neighbors, canonical Watson-Crick base pairs (A-U, G-C), and wobble pairs (G-U). Each edge type receives a learned embedding (dimension=6) that is concatenated with scalar edge attributes – including a binary sequence-vs-pair flag, normalized sequence distance, stem length at both endpoints, loop length at both endpoints, distance to nearest stem-loop junction, estimated pairing energy from nearest-neighbor thermodynamics, and relative distance of both endpoints to the target site – and passed through a small MLP to produce a 16-dimensional edge representation supplied to the GAT layers. Second, we enrich node features beyond the baseline’s 8 dimensions by adding: one-hot encodings of the immediate 5’ and 3’ neighbors (10 dimensions total, capturing trinucleotide context known to influence ADAR selectivity), stem-loop geometry features (stem length, loop length, distance to junction), and estimated base-pairing energy for paired positions. This yields 22-dimensional node features that embed both local sequence motifs and structural stability. Third, we introduce a parallel sequence branch consisting of a 1D convolutional neural network that processes the tokenized RNA sequence independently of the graph: separate 3-mer and 5-mer convolutional filters (48 channels each, with padding to preserve length) capture local motifs, followed by masked max-pooling over sequence positions and projection to a 48-dimensional sequence embedding. Fourth, we optionally incorporate a global attention mechanism: a multi-head self-attention layer (4 heads) operates on the dense node embeddings after GAT processing, computing attention-weighted aggregation across all positions to capture long-range dependencies beyond the graph’s local message-passing horizon, yielding an additional global context vector. Finally, the graph-pooled embedding, sequence CNN embedding, and (if enabled) global attention embedding are concatenated and passed through a two-layer MLP classification head with ReLU activation and dropout. All structural information derives from RNAfold predictions of the secondary structure. The bio-aware model thus integrates graph topology, edge biochemistry, local sequence motifs, structural geometry, and optional global attention into a unified architecture, enabling it to learn multi-scale editing determinants while maintaining full differentiability for end-to-end training.

A critical aspect of both architectures is the position-centric graph encoding scheme. While multiple adenosines within the same duplex share identical RNA sequence and secondary structure, each candidate site generates a distinct graph representation. Node features encoding relative distance from the candidate editing site and the binary target indicator ensure that the graph topology and feature values differ for each evaluated position. Consequently, adenosines from the same duplex appearing in training and validation sets constitute independent examples with non-overlapping representations, preventing information leakage while enabling efficient use of all available editing sites.

### Model selection via F1-optimized threshold search

**Figure 7:**
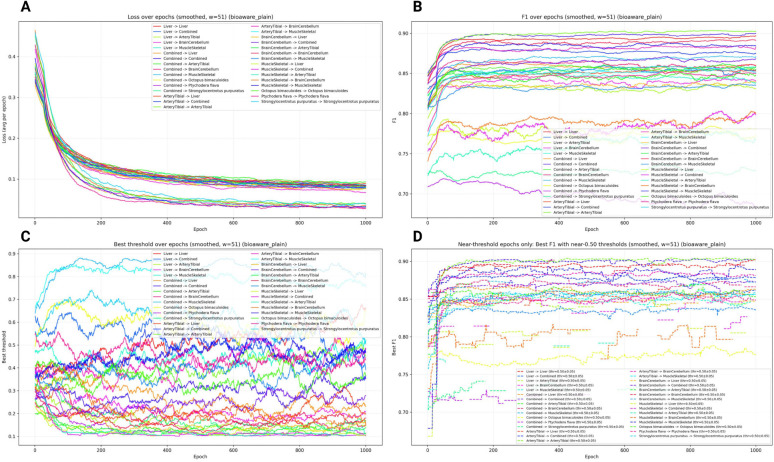
Training dynamics for the bio-aware model across all train→validation settings. Each colored line represents one train→validation tissue or species combination. All curves smoothed with moving average window w=51. (A) Training loss over epochs. (B) Validation F1 score over epochs.(C) Per-epoch optimal decision threshold (threshold maximizing validation F1 at each epoch). (D) Validation F1 over epochs, restricted to epochs where optimal threshold fell within [0.45, 0.55].

To select the best-performing model during training while accounting for the threshold-dependent nature of classification metrics, we employed an F1-optimized threshold search strategy. After each training epoch, we evaluated the model on the held-out validation set and computed performance metrics across a dense grid of 33 decision thresholds, linearly spaced from 0.1 to 0.9. For each threshold candidate, we calculated five classification metrics: accuracy, F1-score, precision (positive predictive value), recall (sensitivity), and specificity (true negative rate). Among all threshold-epoch combinations evaluated during training, we selected the model checkpoint that achieved the maximum F1-score, as this metric optimally balances precision and recall – a critical consideration for RNA editing prediction where both false positives (mispredicted editing sites) and false negatives (missed genuine sites) carry biological and therapeutic consequences. This threshold-adaptive selection procedure was applied independently to each train→validation experiment, identifying the optimal operating point for each tissue or species context. This validation-tuned selection reports an operating point aligned with F1 on the held-out validation split; we therefore treat these metrics as validation estimates rather than unbiased test performance. For threshold-independent evaluation, we additionally computed AUROC (area under the receiver operating characteristic curve) and AUPRC (area under the precision-recall curve), which assess discrimination performance across the entire spectrum of decision boundaries and provide complementary evidence of model quality independent of any specific operating point. In addition, comprehensive analysis of training dynamics across all experimental settings (Appendix C) reveals smooth loss convergence and extended F1 plateaus spanning hundreds of epochs, indicating that top-performing checkpoints occur within stable regimes rather than as isolated fortuitous selections. Notably, performance remains strong even when restricting evaluation to epochs with thresholds near the natural decision boundary (0.45–0.55), confirming that reported results reflect genuine model quality rather than dependence on aggressive threshold optimization (Figure 7).

## 4 Results

### Performance comparison against existing methods

We evaluated both the baseline and bio-aware ADAREDIT architectures against existing state-of-the-art methods to quantify the contribution of graph-based structural representation. To enable direct comparison, we trained both models on the same Liver tissue dataset (5,150 training examples, 1,288 validation examples) used in previous work Rosenwasser et al. (2026) to benchmark three sequence-only approaches: EditPredict (a convolutional neural network), RNA-FM (a pretrained foundation model fine-tuned for editing prediction), and ADAR-GPT (continual fine-tuning of GPT-4o-mini). This benchmark subset is smaller than the full cross-tissue datasets reported below. A critical distinction between ADAREDIT and these prior methods is that sequence-only approaches operate on fixed 201-nucleotide windows without explicit structural encoding, treating dsRNA as a linear string. In contrast, ADAREDIT represents base-pairing relationships as graph edges with graph size adapting to the full duplex structure, enabling the models to learn from the spatial geometry of stem-loop architectures that define ADAR substrate recognition. The baseline ADAREDIT model achieved F1=0.81, accuracy=0.8, AUROC=0.86, and AUPRC=0.85, while the bio-aware model reached F1=0.8, accuracy=0.79, AUROC=0.86, and AUPRC=0.85. Both substantially outperformed all sequence-only baselines: EditPredict (F1=0.72, Δ=+0.08–0.09), RNA-FM (F1=0.71, Δ=+0.09–0.10), and ADAR-GPT (F1=0.76, Δ=+0.04–0.05) (Figure 3A,B). These results demonstrate that explicitly encoding RNA secondary structure alongside sequence information yields substantially higher performance.

### Cross-tissue generalization reveals tissue-specific learning

To systematically assess within-tissue performance and cross-tissue behavior, we trained independent baseline models for each of the 25 train→validation tissue pairs across the five tissue-specific datasets (Liver, Brain Cerebellum, Artery Tibial, Muscle Skeletal, Combined), yielding a 5×5 matrix of 25 train→validation experiments (Figure 3C). In each train→validation setting, we removed any adenosine positions appearing in the training set from the corresponding validation set prior to evaluation. This comprehensive cross-validation design revealed several critical patterns. First, within-tissue performance (diagonal elements of the matrix) consistently achieved the highest F1 scores, with Liver→Liver at F1=0.88, Brain Cerebellum→Brain Cerebellum at F1=0.89, and Artery Tibial→Artery Tibial at F1=0.89, demonstrating that models learn tissue-specific editing patterns when trained and validated on matched ADAR isoform contexts. Second, performance degraded when training and validation sets had divergent ADAR expression profiles: for example, models trained on Liver (ADAR1-dominant) showed reduced performance when validated on Artery Tibial (ADAR2-dominant), with F1 dropping from 0.88 (Liver→Liver) to 0.84 (Liver→Artery Tibial), indicating that substrate preferences differ between ADAR isoforms and that models capture these enzyme-specific determinants. Third, Muscle Skeletal consistently showed the lowest performance both as a training source and validation target, likely reflecting the limited training data (only 5,918 examples) due to low ADAR1 expression and sparse editing activity in this tissue Roth et al. (2019). These cross-tissue experiments demonstrate that the baseline graph architecture successfully learns biologically meaningful patterns.

The bio-aware model, which integrates biochemically-informed features at multiple architectural levels, demonstrated improvements over the baseline graph architecture in the majority of tissue contexts (Figure 3D,E). Beyond the Liver→Liver comparison discussed above (bio-aware F1=0.91 vs baseline F1=0.88, Δ=+0.03), these gains replicated across diverse ADAR isoform environments: Brain Cerebellum→Brain Cerebellum improved from F1=0.89 to F1=0.9 (Δ=+0.01), Artery Tibial→Artery Tibial from F1=0.89 to F1=0.91 (Δ=+0.02), and even the challenging Muscle Skeletal dataset advanced from F1=0.87 to F1=0.91 (Δ=+0.04). The Combined→Combined model achieved the highest overall performance with F1=0.90, accuracy=0.91, AUROC=0.96, and AUPRC=0.96. This architectural enhancement introduces an important trade-off between biological interpretability and model-driven discovery. The baseline model receives minimal explicit guidance – only the graph topology (sequence edges and base-pairing edges) and basic node features (base identity, pairing status, position) – forcing it to learn structural editing determinants entirely from data through the attention mechanism. In contrast, the bio-aware model is provided with extensive prior biological knowledge: typed edges distinguishing canonical Watson-Crick pairs from wobble pairs and sequential connections, with learned edge embeddings and attention weights that dynamically prioritize different edge types; enriched node features encoding 3’ and 5’ neighbors (trinucleotide context known to influence ADAR selectivity), stem-loop geometry (stem length, loop length, distance to junction), and base-pairing energies; plus a parallel sequence CNN branch explicitly designed to capture local motifs. By explicitly encoding these biological features, the bio-aware model is guided toward biochemically meaningful patterns but has less freedom to discover novel structural determinants independently. The predominant pattern of 1–6 percentage point F1 improvements in within-tissue and most cross-tissue contexts demonstrates that explicit biological feature engineering – when grounded in established ADAR biochemistry – generally enhances predictive performance without overfitting to training-set-specific artifacts.

However, the bio-aware model underperforms the baseline in four cross-tissue settings: Liver→Muscle Skeletal (ΔF1=−0.007), Liver→Combined (ΔF1=−0.008), Muscle Skeletal→Artery Tibial (ΔF1=−0.012), and Muscle Skeletal→Liver (ΔF1=−0.005), with one tie in Brain Cerebellum→Liver (ΔF1=0.000). Given the small magnitude of these differences and the limited validation set sizes in some tissues (e.g., Muscle Skeletal *n*=1,480), these cases may reflect statistical variation, overfitting to training-tissue-specific patterns due to increased model complexity, or genuine differences in how engineered structural features transfer across divergent ADAR expression contexts.

### Cross-species generalization suggests partially conserved structural principles

To assess whether ADAREDIT’s graph-based structural encoding captures editing determinants beyond human *Alu* elements, we evaluated models on three evolutionarily distant species lacking *Alu*: sea urchin (S. purpuratus), acorn worm (P. flava), and octopus (O. bimaculoides). These taxa span hundreds of millions of years of divergence and represent independent evolutionary trajectories of A-to-I editing, with editing occurring predominantly in species-specific repetitive elements rather than primate-derived SINEs. Critically, when models were trained and validated on these non-*Alu* datasets, both architectures achieved high performance comparable to human *Alu* results: bio-aware models reached F1=0.875 (sea urchin), F1=0.878 (octopus), and F1=0.865 (acorn worm), while baseline models achieved F1=0.857, F1=0.858, and F1=0.873 respectively (Figure 3F). These within-species results demonstrate that graph-based RNA structure encoding successfully captures ADAR substrate recognition patterns even in non-*Alu* contexts, validating that our approach learns from dsRNA topology rather than *Alu*-specific sequence features. More strikingly, models trained exclusively on human data (Combined dataset) transferred effectively to non-mammalian species: Combined→octopus achieved F1=0.805 (bio-aware) and F1=0.787 (baseline), Combined→sea urchin reached F1=0.762 (bio-aware) and F1=0.745 (baseline), and Combined→acorn worm attained F1=0.750 (baseline) and F1=0.736 (bio-aware) (Figure 3F,G,H). While cross-species performance showed moderate degradation (Δ F1=−0.07 to −0.12), these results far exceeded random chance and demonstrate that structural features learned from human *Alu* duplexes partially generalize to evolutionarily distant organisms. The performance reduction likely reflects genuine biochemical differences in ADAR substrate preferences evolved independently across lineages, while the substantial remaining predictive power indicates partial conservation of structural recognition features – dsRNA stability, structural constraints, and local sequence context – across deep evolutionary time. Comparing bio-aware and baseline across all cross-species settings (Table 1 in Appendix B), bio-aware achieves higher F1 in four of six cases, with baseline performing better in the remaining two. Both models demonstrate effective cross-species transfer.

### Generalization to protein-coding editing sites

To assess whether models trained exclusively on *Alu* elements generalize to functionally important editing sites in protein-coding transcripts, we evaluated performance on seven well-characterized coding and regulatory sites across diverse genes: AJUBA, BLCAP, FLNA, TTYH2, NEIL1 (primarily ADAR1 substrates), and GRIA2, GRIA3 (primarily ADAR2 substrates), representing both constitutive and tissue-specific editing events (Figure 4). These targets constitute a held-out data distribution entirely absent from training, providing an out-of-distribution test of model generalization. For each gene, we computationally identified the dsRNA structure encompassing the edited region by extracting transcript sequences flanking the target site and predicting secondary structure using RNAfold. We then catalogued all adenosines within these predicted dsRNA regions and classified them into two categories based on GTEx experimental editing data from three tissue contexts (Brain Cerebellum, Liver, Combined): edited sites (>15% editing) and non-edited sites (<1% editing), creating evaluation sets for each gene-tissue combination. For each tissue context, we applied the corresponding Alu-trained model – baseline (Figure 4A) or bio-aware (Figure 4B) models trained on Brain Cerebellum *Alu*, Liver *Alu*, or Combined *Alu* datasets – to predict editing probability for each adenosine.

Baseline model performance (Figure 4A) showed strong separation for several ADAR1 targets. AJUBA exhibited excellent discrimination across all three tissue contexts, with edited sites showing median prediction scores >0.8 and non-edited sites <0.2. BLCAP showed very strong separation in Brain and Combined datasets with edited sites reaching median ≈0.95 and non-edited sites near 0.3 and 0.05 respectively, though Liver showed weaker separation with considerable overlap. FLNA demonstrated strong separation with edited sites median ≈0.95–1.0 and non-edited near 0. TTYH2 showed significant separation in Combined with edited sites showing median ≈1 and non-edited sites near 0, but no separation in Brain and Liver (ns). NEIL1 showed no statistically significant separation across all three tissues, though visual separation was observed in Combined and Liver. For ADAR2 targets GRIA2 and GRIA3, both genes showed variable performance across tissues: no significant separation in Brain, but modest to moderate separation in Combined and Liver.

Bio-aware model performance (Figure 4B) generally showed similar or slightly improved patterns. AJUBA maintained excellent separation. BLCAP showed strong separation in Brain, but weaker in Combined and Liver. FLNA demonstrated consistent separation across tissues with edited sites median 0.9–1. TTYH2 and NEIL1 showed no separation across all tissues. GRIA2 showed strong separation specifically in Brain, but no separation in Combined and Liver. GRIA3 showed no significant separation across all three tissues.

An illustrative example from the FLNA target (Figure 4C) shows the full dsRNA structure with predictions from both baseline (top) and bio-aware (bottom) models overlaid on experimental editing annotations, demonstrating how both architectures correctly identify the majority of edited sites while maintaining specificity.

Overall, the models demonstrated differential generalization across target types. ADAR1-dominant targets (AJUBA, BLCAP, FLNA) showed consistently strong performance in both baseline and bio-aware models, validating that graph-based structural encoding captured transferable ADAR1 substrate recognition principles beyond *Alu*-specific patterns. In contrast, ADAR2 targets showed weaker and more variable performance. Notably, GRIA2 achieved strong discrimination only in the Brain-trained bio-aware model, reflecting ADAR2’s tissue-specific expression and suggesting partial capture of ADAR2-specific biochemical rules through brain-matched training data. This ADAR1-versus-ADAR2 asymmetry aligns with training data composition: *Alu* elements are predominantly ADAR1 substrates, leading models to preferentially learn ADAR1 recognition rules while underrepresenting ADAR2’s distinct preferences.

## 5 Model interpretability

### Interpretability through attention analysis of the baseline model

A central motivation for employing graph attention networks in ADAREDIT was to combine high predictive accuracy with biological interpretability. Graph attention mechanisms offer a framework for interpretability by revealing which neighboring nodes receive high weights during prediction Knyazev et al. (2019); Brody et al. (2021). While these patterns reflect learned associations rather than proven causal relationships, they can guide hypothesis generation when patterns align with established biochemistry. To understand what structural and sequence patterns the model learns independently – without explicit biological guidance – we performed all interpretability analyses on the baseline architecture rather than the bio-aware variant. The baseline model receives only graph topology (sequence edges and base-pairing edges) and minimal node features (base identity, pairing status, position), forcing it to discover editing determinants entirely from data through the attention mechanism. By analyzing baseline attention weights, we can identify which positional and structural features the model autonomously prioritizes, offering unbiased insights into learned editing determinants.

We conducted two complementary interpretability approaches (Figure 5A). First, to validate that attention weights contain genuine predictive signal rather than spurious patterns, we extracted edge-level attention coefficients from the first GAT layer and aggregated them into positional features representing the maximum attention at each position in a ±50 nucleotide window centered on the candidate editing site. These attention-derived positional features were then used to train an independent XGBoost classifier Chen & Guestrin (2016), providing a supervised assessment of whether attention alone captures editing determinants Belinkov (2022). To identify which specific positions contribute most to predictions, we applied SHAP (SHapley Additive exPlanations) analysis Lundberg & Lee (2017) to the XGBoost model, ranking features by their impact on model output. We then retrained the XGBoost classifier using only the top 20 SHAP-identified features and repeated SHAP analysis to confirm that this reduced feature set retained interpretability. Second, to directly examine attention patterns without supervised intermediaries, we performed descriptive statistical analyses of mean attention across the ±50 nucleotide window, stratifying by editing status (edited vs non-edited sites), nucleotide identity (A, U, G, C at each position), and structural context (base-paired vs unpaired/loop positions).

The supervised validation demonstrated that attention weights encode substantial predictive information (Figure 5B). Remarkably, an XGBoost classifier trained solely on attention-derived positional features – without access to the original sequence, structure, or any engineered features – achieved strong performance on the Combined dataset: F1=0.82, accuracy=0.81, precision=0.80, and recall=0.83. These robust results demonstrate that attention coefficients alone capture the majority of predictive signal, consistent with the hypothesis that they reflect interpretable positional patterns rather than serving as computational artifacts. As expected, the full baseline model achieved higher performance (F1=0.89, accuracy=0.89), as it leverages additional information from multi-layer graph representations, node embeddings after message passing, and global pooling operations. However, the ability of attention weights alone to achieve F1=0.82 is consistent with the hypothesis that the model’s decision-making process reflects interpretable positional patterns. SHAP analysis of the XGBoost model (Figure 5C) revealed that positions immediately surrounding the editing site (positions 0, ±1, ±2) ranked as the most influential features, aligning with experimental findings identifying these nucleotides as critical determinants of editing efficiency. When retrained using only the top 20 SHAP-identified features, the XGBoost model maintained nearly identical performance (F1=0.82, accuracy=0.81; Figure 5B), with SHAP values again highlighting the same proximal positions (Figure 5D), confirming that a small, interpretable subset of positional attention features retains full attention-based predictive power and facilitates biological interpretation.

Descriptive analysis of attention patterns revealed that the baseline model’s learned representations are consistent with established biochemical rules. Global attention profiles (Figure 5E) showed that the editing site (position 0) receives substantially higher mean attention than flanking regions for both edited sites (orange line) and non-edited sites (cyan line), with non-edited sites exhibiting slightly elevated attention across the entire ±50 nucleotide window. Stratifying attention by nucleotide identity (Figure 5F) revealed a striking pattern: position −1 exhibits the highest attention when occupied by guanosine (G, red line), consistent with the well-established 5’ neighbor preference rule where upstream G suppresses editing – demonstrating that the model autonomously discovered this critical biochemical constraint without explicit instruction. Structural context analysis (Figure 5G) demonstrated that unpaired/loop positions (cyan line) receive markedly higher mean attention than base-paired positions (purple line) throughout the window, with a dramatic attention peak at the editing site itself (position 0).

### In silico mutagenesis reveals sequence and structure preferences at core editing positions

To systematically probe the baseline model’s learned sequence and structural preferences, we performed comprehensive in silico mutagenesis analysis on high-confidence edited sites (model prediction > 0.7 on true edited sites from the validation set). For each edited site, we generated all possible single-nucleotide variants at positions spanning from −3 to +3 relative to the editing site (position 0), maintaining the target adenosine unchanged. At each position, we substituted the original nucleotide with each of the four bases (A, G, C, T/U), updated the corresponding node features in the graph representation, and measured the change in model prediction. To isolate sequence effects from structural effects, we performed two parallel analyses: (1) sequence-only mutagenesis, where only the base identity was altered while preserving the original pairing status, and (2) structure-coupled mutagenesis, where mutations that would disrupt canonical or wobble base pairs (A–U/T, G–C, G–U/T) also triggered structural changes by modifying the paired/unpaired status and removing the corresponding base-pairing edge from the graph. We aggregated results across analyzed edited sites to generate mean preference scores for each base at each position, normalized by subtracting the position-wise mean to highlight relative preferences (Figure 6A).

The sequence preference analysis (Figure 6A) revealed striking patterns consistent with established ADAR biochemistry. At position −1 (5’ neighbor), the model showed the strongest preference for U (T), with substantially reduced predictions for G, recapitulating the well-documented 5’ neighbor preference rule where upstream purines – particularly G – strongly suppress editing Polson & Bass (1994); Eggington et al. (2011). This suppression extended to position −2, where G also showed reduced preference compared to pyrimidines. Conversely, at position +1 (3’ neighbor), the model exhibited a preference for G, aligning with experimental observations that 3’ purines can enhance editing efficiency Eggington et al. (2011). At the editing site itself (position 0), only adenosine is biologically relevant, so this position was excluded from base-substitution analysis.

The structure-coupled mutagenesis analysis (Figure 6B) revealed distinct pairing preferences that vary by both position and nucleotide identity. At position −1, the model exhibited a striking base-specific structural preference: when guanosine occupies this position, the model strongly prefers it to remain unpaired (positive values in heatmap indicate preference for paired status; negative values indicate preference for unpaired status), consistent with structural studies showing that unpaired G at −1 facilitates ADAR binding Doherty et al. (2022). In contrast, when position −1 contains U, C, or A, the model showed a moderate preference for these bases to be paired, suggesting that structural context requirements differ based on local sequence composition. At position +1, pyrimidines (U and C) exhibited strong preferences to be base-paired, potentially reflecting a requirement for structural stability in the 3’ stem region to properly position the editing site for catalysis. These position- and base-specific structural preferences demonstrate that the model has learned coupled sequence-structure rules rather than treating structure as a position-independent feature.

To examine structural preferences beyond the immediate editing motif, we performed positional structural perturbation analysis across a broad window spanning positions −40 to +40 (Figure 6C). For each position, we compared model predictions when the nucleotide was forced to be base-paired (edge retained in graph) versus unpaired (pairing edge removed from graph), measuring the impact as the difference: Paired minus Unpaired. Positive values indicate positions where base-pairing enhances editing probability, while negative values indicate positions where unpaired/loop configurations are preferred. The analysis revealed several striking features (Figure 6C). First, position 0 (the editing site) showed the strongest preference to be unpaired, with a large negative impact score, confirming the fundamental requirement for adenosine accessibility. Second, positions ~30–35 downstream of the editing site exhibited a significant preference to be unpaired, aligning with previous studies Zambrano-Mila et al. (2023). Third, positions around +20 showed a preference to be paired, potentially reflecting a requirement for a stable stem structure in the mid-3’ region to properly scaffold the editing site. Fourth, the overall signal was substantially stronger and more consistent in the downstream (3’) region compared to upstream (5’), suggesting that 3’ structural features exert greater influence on ADAR substrate recognition and catalysis. This asymmetry may reflect the directional nature of ADAR binding and scanning along dsRNA substrates. The ability of the model to autonomously identify these positional structural preferences suggests that graph-based encoding enables learning of long-range structural determinants beyond local sequence context.

To directly assess whether the model learned canonical base-pairing rules and their influence on editing, we performed a base-pairing interaction analysis at the three core positions (-1, 0, +1) (Figure 6D). For each position, we systematically varied both the sequence-side nucleotide and its opposing partner nucleotide across all 16 possible combinations (4 bases × 4 bases), then evaluated the structural validity of each pair. Valid pairs (Watson-Crick: A–U/T, G–C; wobble: G–U/T) were marked as paired (feature value = 1) with the base-pairing edge retained in the graph, while invalid pairs triggered structural disruption: the pairing status was set to 0 and the base-pairing edge was removed from the graph to reflect the absence of stable hydrogen bonding. This topology-aware perturbation allowed us to test whether the model responds appropriately to both sequence changes and their structural consequences. Results are presented as 4×4 heatmaps where rows represent the sequence-side base and columns represent the opposing partner base, with prediction scores indicating editing probability (Figure 6D).

At position 0 (the editing site), the strongest prediction occurred when adenosine (A) was opposed by cytosine (C), recapitulating the well-established A:C mismatch preference of ADAR enzymes Wong et al. (2001). At position −1, the model showed elevated predictions when guanosine (G) was opposed by guanosine (G), forming a G:G mismatch, consistent with previous studies demonstrating that G:G mismatches at −1 enhance editing by creating local structural distortion that facilitates ADAR binding Doherty et al. (2022). Conversely, when position −1 contained uracil (U/T) with guanosine (G) opposite, predictions were substantially reduced, matching experimental findings that U:G wobble pairs at −1 suppress editing and can be exploited for site-directed RNA editing control Reautschnig et al. (2025). More broadly, position +1 showed a general preference for stable base-pairing (strong signals along the Watson-Crick diagonal: A:T, G:C), reflecting the requirement for structural integrity in the 3’ stem to position the editing site correctly. Together, these interaction analyses demonstrate that the baseline model autonomously learned not only individual base preferences but also context-dependent base-pairing rules, consistent with the hypothesis that graph-based structural encoding captures the coupled sequence-structure logic governing ADAR substrate recognition.

## 6 DISCUSSION

ADAREDIT demonstrates that integrating RNA secondary structure explicitly into predictive models of A-to-I editing substantially improves both accuracy and biological interpretability. Unlike sequence-based or structure-implicit approaches, our graph-based framework captures the spatial rules that govern ADAR substrate recognition – such as position-specific pairing, local asymmetry, and inhibitory neighbor bases – without requiring manual feature design.

Beyond predictive performance, ADAREDIT may offer future value for the design of site-directed RNA editing therapies. Recent advances have shown that endogenous ADAR enzymes can be co-opted using synthetic guide RNAs to correct pathogenic mutations. However, efficient guide design remains challenging, often requiring laborious empirical screening. ADAREDIT could potentially address this bottleneck by enabling computational prioritization of candidate guides across the transcriptome. Rather than testing dozens or hundreds of guide configurations in vitro, researchers can use the model to rapidly score large design spaces and focus experimental validation on the most promising candidates – reducing cost, time, and design uncertainty.

While the model generalizes well across tissues and species, its performance on certain ADAR2-dominant targets remains modest. This likely reflects the composition of the training data: ADAREDIT was trained primarily on *Alu* elements, which are abundant in the human transcriptome and predominantly edited by ADAR1. Expanding the training set to include a broader repertoire of coding-region edits and validated ADAR2 substrates will likely improve the model’s sensitivity to isoform-specific rules. The framework itself is agnostic to ADAR subtype, and can naturally incorporate such data as it becomes available.

Looking ahead, models like ADAREDIT could help shift RNA editing design from empirical trial-and-error toward predictive, computation-guided strategies. By enabling transcriptome-scale prioritization of candidate sites and guide configurations, such models can streamline experimental workflows and accelerate therapeutic development. As the field progresses, incorporating cell-type context, isoform preferences, and experimentally measured RNA structures may further improve prediction accuracy and broaden clinical applicability.

### Scope and limitations

#### Scope.

ADAREDIT is designed for predicting A-to-I RNA editing sites in double-stranded RNA contexts where ADAR enzyme recognition depends on both sequence and secondary structure features. The model operates on computationally predicted RNA secondary structures (RNAfold) representing dsRNA duplexes. It has been trained and validated across five human tissues with diverse ADAR expression profiles (Brain Cerebellum, Artery Tibial, Liver, Muscle Skeletal, Combined) and three evolutionarily distant non-mammalian species lacking *Alu* elements (sea urchin, acorn worm, octopus). The framework is intended for transcriptome-scale prioritization of candidate editing sites, mechanistic interpretation of structural and sequence determinants governing ADAR specificity, and computational pre-screening for guide RNA design in site-directed editing applications. While the current implementation focuses on *Alu*-derived duplexes and selected protein-coding targets in human tissues, the graph-based architecture is generalizable to any dsRNA context where secondary structure can be predicted or experimentally determined.

#### Limitations.

Several technical and biological limitations warrant consideration. First, ADAREDIT relies entirely on computationally predicted secondary structures from RNAfold minimum free energy models, which may not capture dynamic conformational ensembles or tertiary interactions that influence editing. Second, the model does not account for additional RNA-binding proteins that can compete with or facilitate ADAR binding to substrates, thereby modulating editing levels in cellular contexts. Third, binary classification (edited vs. non-edited) does not provide quantitative prediction of editing stoichiometry – a limitation for therapeutic applications requiring precise dosage control. Fourth, interpretability analyses via attention weights, SHAP, and in silico mutagenesis provide hypothesis-generating signals about which features the model uses for prediction, but they do not establish causality or direct molecular mechanisms. We therefore frame these analyses as consistency checks against known ADAR biochemical preferences rather than as definitive proof of mechanism, and experimental validation is required to confirm the biological relevance of identified patterns and rule out spurious correlations. Finally, the model has been evaluated primarily within the *Alu*-derived duplex paradigm, and broader validation across diverse non-*Alu* substrates, alternative splicing isoforms, and species-specific editing contexts remains an important direction for future development.

### Data, Materials, and Software Availability

All code, trained models, and datasets are publicly available at: https://github.com/Scientific-Computing-Lab/AdarEdit. The repository includes implementations of baseline and bio-aware graph attention architectures, training and evaluation scripts, interpretability analysis tools (SHAP, attention visualization, in silico mutagenesis), and dataset partitions for all tissue and species experiments. Human tissue datasets were derived from 905 *Alu* pairs with editing levels from GTEx v7 RNA-seq data. Cross-species datasets were constructed from editing sites reported in Zhang et al Zhang et al. (2023). All RNA secondary structures were predicted using RNAfold (ViennaRNA Package).

#### LLM Usage.

In preparing this paper, the LLM was used only for writing and editing, and it does not impact the core methodology, experiments, or results. The authors reviewed the LLM-assisted edits and take full responsibility for the content.

## A Previous Work on A-to-I RNA Editing Prediction

Computational prediction of A-to-I editing sites has progressed through several methodological phases, reflecting a steady shift from manually designed descriptors to representation learning. Early predictors relied on classical machine learning models (e.g., support vector machines, random forests, and XGBoost) built on handcrafted features such as local sequence motifs, nucleotide composition around candidate sites, thermodynamic properties derived from RNA secondary structure, and evolutionary conservation scores Tac et al. (2021); Ouyang et al. (2018); Liu et al. (2021); Jiang et al. (2024); Chen et al. (2016). Although these approaches achieved moderate accuracy, their reliance on predefined biological descriptors constrained the discovery of editing determinants outside the chosen feature set and often required substantial manual reengineering when transferred across datasets or conditions. Recent work continues to explore combinations of sequence and secondary-structure descriptors in broader evaluation settings, including cross-testing and conservation-oriented analyses Zawisza-Álvarez et al. (2024).

With the rise of deep learning, convolutional and recurrent architectures began to learn predictive patterns directly from sequence windows, reducing dependence on explicit feature engineering. EditPredict Wang et al. (2021); Ouyang et al. (2018) uses convolutional networks on 201-nucleotide windows to classify editing sites, while other frameworks combine multiple sequence-derived views or extend prediction to alternative sequencing modalities (e.g., nanopore) Chen et al. (2023b,a). More recently, PreAIS Fang et al. (2025) further illustrates the continued strength of deep learning for direct site prediction using DNN/CNN-based modeling and modern interpretability analyses. Despite these advances, most deep learning predictors still treat RNA primarily as a linear string, and structural context is typically incorporated only indirectly (as derived features) rather than encoded as an explicit relational substrate.

Large pre-trained models have also been adapted to editing prediction, motivated by their ability to capture richer contextual dependencies. RNA-FM Shen et al. (2024), trained on millions of RNA sequences with a masked-language objective, can be fine-tuned for downstream editing tasks. ADAR-GPT Rosenwasser et al. (2026) applies continual fine-tuning of GPT-4o-mini on sequence windows and shows competitive performance when benchmarked against established deep learning baselines. In a related therapeutic setting, Helix Cao et al. (2025) augments transformer attention with base-pairing probability matrices to predict on-target editing efficiency for guide RNA design. In parallel, complementary lines of work focus on improving the reliability of editing labels and calling editing events directly from RNA-seq, including deep learning frameworks designed to separate RNA editing from DNA variation Fu et al. (2024) and temporal convolutional classifiers trained on large collections of known A-to-I events Fonzino et al. (2025). These efforts are valuable for curating high-confidence training sets, but they do not directly address the core representational challenge of encoding dsRNA geometry as a native relational structure for site-level prediction.

Graph neural networks have been widely used to model RNA structure and function in adjacent domains, including RNA secondary structure learning Zhang et al. (2021), RNA–protein interaction prediction Yamada & Hamada (2022); Saon et al. (2024), and hybrid architectures that integrate language model embeddings with graph attention for binding-site tasks Xiao et al. (2024). Structure-aware non-GNN predictors have also incorporated RNAfold-derived base-pairing information into editing classification Liu et al. (2021). However, A-to-I editing-site predictors remain overwhelmingly sequence-centric – CNNs, RNNs, transformers, or feature-based models – rather than graph-native architectures that explicitly encode both sequential adjacency and base-pairing relationships within a unified representation. We are not aware of prior graph-based predictors purpose-built for A-to-I editing-site classification that natively integrate these edge types while providing attention-level interpretability aligned with established ADAR biochemistry. This positions ADAREDIT in a distinct methodological space relative to existing editing predictors and the broader GNN-for-RNA literature.

## B Comprehensive Performance Metrics Across All Experimental Settings

Table 1 presents performance metrics across all 31 train→validation experiments: 25 cross-tissue combinations spanning five human tissue contexts with distinct ADAR expression profiles, and six cross-species evaluations including three evolutionarily distant non-*Alu* organisms. For each experimental setting, we report dataset sizes, classification metrics (F1, accuracy, precision, recall, specificity) at the optimal decision threshold, and the superior architecture (Bio-aware vs. Baseline). Across these diverse evaluation contexts, bio-aware models consistently outperform baseline architectures in 20 of 25 tissue combinations and 4 of 6 cross-species settings, demonstrating that explicit encoding of biochemical constraints enhances generalization while retaining the capacity for data-driven discovery.

## C Training Dynamics and Checkpoint Robustness

We provide a comprehensive characterization of training behavior for the bio-aware architecture across all train→validation experimental settings (Figure 7). These analyses address potential concerns that F1-optimized checkpoint selection might reflect fortuitous identification of isolated high-performing epochs rather than stable converged solutions. Training loss trajectories (Figure 7A) decrease monotonically from initial values toward clear asymptotic plateaus across all experimental settings, with no evidence of late-epoch instability or divergence, indicating well-behaved optimization dynamics under our training protocol. Validation F1 scores (Figure 7B) rise rapidly during early training epochs and subsequently maintain consistently high performance across extended temporal regimes, typically spanning several hundred epochs. Crucially, top-performing epochs reported in our results occur within broad stable plateaus characterized by narrow-band fluctuations rather than as isolated transient peaks. This plateau structure indicates that checkpoint selection within these regimes yields effectively equivalent performance, supporting robustness to the specific epoch chosen.

Per-epoch optimal decision thresholds (Figure 7C) exhibit brief initial adjustment during early training, followed by smooth, gradual evolution around stable operating bands. Notably, optimal thresholds show no abrupt discontinuities or isolated spikes in later training phases, indicating that the learned decision boundary stabilizes as optimization progresses. To evaluate sensitivity to threshold selection, we computed F1 trajectories using only epochs where optimal thresholds fell within [0.45, 0.55] (Figure 7D), corresponding to operating points near the natural decision boundary for balanced datasets. These constrained trajectories exhibit plateau characteristics qualitatively and quantitatively comparable to unconstrained analysis, demonstrating that reported performance is not contingent on selection of extreme threshold values. These training characteristics replicate consistently across within-tissue, cross-tissue, and cross-species evaluation contexts, supporting the interpretation that reported validation metrics reflect stable learned representations rather than artifacts of optimization or selection procedures.

**Table 1:** Comprehensive performance metrics including dataset sizes. Comparison of Bio-aware vs. Baseline models across all contexts. *N_train_* and *N_val_* denote dataset sizes. **Winner** is determined by the highest F1 score.

Context		Size (*N*)		F1 Score		Accuracy		Precision		Recall		Specificity		Winner
						
Train Set	Val Set	Train	Val	Bio	Base	Bio	Base	Bio	Base	Bio	Base	Bio	Base	

Artery Tibial	Artery Tibial	19.2k	4.8k	**0.912**	0.888	**0.910**	0.885	**0.900**	0.866	**0.923**	0.911	**0.898**	0.858	**Bio**
	Brain Cer.	19.2k	4.8k	**0.897**	0.882	**0.895**	0.878	**0.883**	0.852	0.911	**0.914**	**0.879**	0.841	**Bio**
	Liver	19.2k	3.9k	**0.867**	0.852	**0.862**	0.849	0.834	**0.836**	**0.904**	0.869	0.820	**0.829**	**Bio**
	Muscle Skel.	19.2k	1.5k	**0.870**	0.864	**0.865**	0.861	0.841	**0.843**	**0.901**	0.886	0.830	**0.835**	**Bio**
	Combined	19.2k	4.8k	**0.892**	0.880	**0.889**	0.878	**0.872**	0.869	**0.912**	0.891	0.865	**0.865**	**Bio**

Brain Cer.	Artery Tibial	19.2k	4.8k	**0.877**	0.868	**0.876**	0.864	**0.869**	0.842	0.885	**0.897**	**0.866**	0.830	**Bio**
	Brain Cer.	19.2k	4.8k	**0.904**	0.888	**0.904**	0.885	**0.906**	0.862	0.903	**0.917**	**0.906**	0.853	**Bio**
	Liver	19.2k	3.9k	0.858	0.858	**0.852**	0.851	**0.830**	0.822	0.888	**0.897**	**0.817**	0.805	–
	Muscle Skel.	19.2k	1.5k	**0.856**	0.854	**0.849**	0.844	**0.820**	0.800	0.895	**0.916**	**0.804**	0.771	**Bio**
	Combined	19.2k	4.8k	**0.882**	0.879	**0.884**	0.875	**0.896**	0.851	0.869	**0.910**	**0.899**	0.840	**Bio**

Liver	Artery Tibial	15.7k	4.8k	**0.854**	0.844	**0.846**	0.837	0.813	**0.814**	**0.899**	0.875	0.793	**0.799**	**Bio**
	Brain Cer.	15.7k	4.8k	**0.861**	0.856	**0.856**	0.848	**0.836**	0.814	0.888	**0.902**	**0.825**	0.794	**Bio**
	Liver	15.7k	3.9k	**0.906**	0.879	**0.905**	0.878	**0.902**	0.866	**0.910**	0.893	**0.901**	0.862	**Bio**
	Muscle Skel.	15.7k	1.5k	0.869	**0.876**	0.865	**0.870**	**0.848**	0.842	0.890	**0.912**	**0.840**	0.828	Base
	Combined	15.7k	4.8k	0.876	**0.884**	0.870	**0.878**	0.842	**0.843**	0.912	**0.930**	**0.829**	0.827	Base

Muscle Skel.	Artery Tibial	5.9k	4.8k	0.818	**0.830**	0.807	**0.821**	0.774	**0.789**	0.867	**0.876**	0.746	**0.765**	Base
	Brain Cer.	5.9k	4.8k	**0.829**	0.815	**0.821**	0.804	**0.798**	0.773	**0.862**	0.862	**0.780**	0.746	**Bio**
	Liver	5.9k	3.9k	0.823	**0.828**	**0.825**	0.813	**0.833**	0.768	0.813	**0.896**	**0.837**	0.728	Base
	Muscle Skel.	5.9k	1.5k	**0.908**	0.871	**0.906**	0.867	**0.893**	0.844	**0.923**	0.900	**0.889**	0.833	**Bio**
	Combined	5.9k	4.8k	**0.840**	0.835	**0.836**	0.821	**0.819**	0.774	0.862	**0.908**	**0.809**	0.734	**Bio**

Combined	Artery Tibial	19.2k	4.8k	**0.875**	0.848	**0.873**	0.840	**0.861**	0.810	**0.890**	0.889	**0.856**	0.792	**Bio**
	Brain Cer.	19.2k	4.8k	**0.880**	0.870	**0.877**	0.867	**0.862**	0.848	**0.899**	0.893	**0.855**	0.840	**Bio**
	Liver	19.2k	3.9k	**0.877**	0.821	**0.872**	0.806	**0.848**	0.762	**0.907**	0.890	**0.837**	0.721	**Bio**
	Muscle Skel.	19.2k	1.5k	**0.889**	0.868	**0.886**	0.863	**0.870**	0.836	**0.910**	0.903	**0.863**	0.823	**Bio**
	Combined	19.2k	4.8k	**0.908**	0.889	**0.907**	0.887	**0.895**	0.874	**0.922**	0.904	**0.891**	0.869	**Bio**

*Cross-Species Generalization*														

Combined	*S. purp.*	19.2k	1.5k	**0.762**	0.745	**0.754**	0.713	**0.706**	0.645	0.826	**0.882**	**0.688**	0.559	**Bio**
Combined	*P. flava*	19.2k	1.5k	0.736	**0.750**	0.703	**0.717**	0.659	**0.669**	0.835	**0.852**	0.573	**0.585**	Base
Combined	*O. bimac.*	19.2k	1.5k	**0.805**	0.787	**0.788**	0.748	**0.745**	0.683	0.875	**0.928**	**0.700**	0.567	**Bio**
*S. purp.*	*S. purp.*	6.1k	1.5k	**0.875**	0.857	**0.879**	0.854	**0.860**	0.801	0.891	**0.921**	**0.868**	0.792	**Bio**
*P. flava*	*P. flava*	6.1k	1.5k	0.864	**0.873**	0.862	**0.870**	0.844	**0.851**	0.886	**0.896**	0.838	**0.845**	Base
*O. bimac.*	*O. bimac.*	6.1k	1.5k	**0.878**	0.858	**0.877**	0.853	**0.870**	0.828	0.887	**0.891**	**0.867**	0.815	**Bio**
